# Combined cognitive and vocational interventions after mild to moderate traumatic brain injury: study protocol for a randomized controlled trial

**DOI:** 10.1186/s13063-017-2218-7

**Published:** 2017-10-17

**Authors:** Emilie I. Howe, Knut-Petter S. Langlo, Hans Christoffer Aargaard Terjesen, Cecilie Røe, Anne-Kristine Schanke, Helene L. Søberg, Unni Sveen, Eline Aas, Heidi Enehaug, Daniele E. Alves, Pål Klethagen, Kjersti Sagstad, Christine M. Moen, Karin Torsteinsbrend, Anne-Margrethe Linnestad, Tonje Haug Nordenmark, Birte Sand Rismyhr, Grete Wangen, Juan Lu, Jennie Ponsford, Elizabeth W. Twamley, Helene Ugelstad, Øystein Spjelkavik, Marianne Løvstad, Nada Andelic

**Affiliations:** 1 Department of Physical Medicine and Rehabilitation, Oslo University Hospital, Oslo, Norway; 2 Institute of Clinical Medicine, Faculty of Medicine, University of Oslo, Oslo, Norway; 3 Department of Psychology, Faculty of Social Sciences, University of Oslo, Oslo, Norway; 4 The Work Research Institute, Oslo and Akershus University College of Applied Science, Oslo, Norway; 5 Department of Research, Sunnaas Rehabilitation Hospital, Nesoddtangen, Norway; 6 Faculty of Health Sciences, Oslo and Akershus University College of Applied Science, Oslo, Norway; 7 Department of Health Economics, Institute of Health and Society, Faculty of Medicine, University of Oslo, Oslo, Norway; 8 Department of Vocational Rehabilitation, Norwegian Labor and Welfare Administration, Oslo, Norway; 9 The Norwegian User Organization (Personskadeforbundet LTN), Oslo, Norway; 10 Department of Family Medicine and Population Health, Division of Epidemiology, Virginia Commonwealth University, Richmond, VA USA; 11 School of Psychological Sciences and Monash Institute of Cognitive and Clinical Neurosciences, Monash University, Faculty of Medicine, Nursing and Health Sciences , Clayton, Victoria, Australia; 12 Center of Excellence for Stress and Mental Health, VA San Diego Healthcare System, San Diego, CA USA; 13 Department of Psychiatry, University of California, San Diego, San Diego, CA USA; 14 Center for Habilitation and Rehabilitation Models and Services (CHARM), Institute of Health and Society, Faculty of Medicine, University of Oslo, Oslo, Norway

**Keywords:** Mild traumatic brain injury, Cognitive remediation, Supported employment, Individual Placement and Support (IPS), Five-Step Process, Return to work, Work inclusion, Disability management

## Abstract

**Background:**

A considerable proportion of patients with mild to moderate traumatic brain injury (TBI) experience long-lasting somatic, cognitive, and emotional symptoms that may hamper their capacity to return to work (RTW). Although several studies have described medical, psychological, and work-related factors that predict RTW after TBI, well-controlled intervention studies regarding RTW are scarce. Furthermore, there has traditionally been weak collaboration among health-related rehabilitation services, the labor and welfare sector, and workplaces.

**Methods/design:**

This study protocol describes an innovative randomized controlled trial in which we will explore the effect of combining manualized cognitive rehabilitation (Compensatory Cognitive Training [CCT]) and supported employment (SE) on RTW and related outcomes for patients with mild to moderate TBI in real-life competitive work settings. The study will be carried out in the southeastern region of Norway and thereby be performed within the Norwegian welfare system. Patients aged 18–60 years with mild to moderate TBI who are employed in a minimum 50% position at the time of injury and sick-listed 50% or more for postconcussive symptoms 2 months postinjury will be included in the study. A comprehensive assessment of neurocognitive function, self-reported symptoms, emotional distress, coping style, and quality of life will be performed at baseline, immediately after CCT (3 months after inclusion), following the end of SE (6 months after inclusion), and 12 months following study inclusion. The primary outcome measures are the proportion of participants who have returned to work at 12-month follow-up and length of time until RTW, in addition to work stability as well as work productivity over the first year following the intervention. Secondary outcomes include changes in self-reported symptoms, emotional and cognitive function, and quality of life. Additionally, a qualitative RTW process evaluation focused on organizational challenges at the workplace will be performed.

**Discussion:**

The proposed study will combine cognitive and vocational rehabilitation and explore the efficacy of increased cross-sectoral collaboration between specialized health care services and the labor and welfare system. If the intervention proves effective, the project will describe the cost-effectiveness and utility of the program and thereby provide important information for policy makers. In addition, knowledge about the RTW process for persons with TBI and their workplaces will be provided.

**Trial registration:**

ClinicalTrials.gov, NCT03092713. Registered on 10 March 2017.

**Electronic supplementary material:**

The online version of this article (doi:10.1186/s13063-017-2218-7) contains supplementary material, which is available to authorized users.

## Background

Successful return to work (RTW) is a major challenge after traumatic brain injury (TBI) [1–6]. Personal factors such as educational level and occupational status, as well as injury-related characteristics, may predict vocational outcome [7–9]. The person’s own perceptions and motivations regarding RTW, as well as aspects of the workplace environment, also have been associated with RTW and work participation after sickness-related absence [10, 11]. Existing literature suggests that the proportion of individuals with TBI who return to work varies from 13 to 85% [7, 8, 12]. Many TBI survivors return to work prematurely and fail to cope with work demands over time once the full impact of the injury is realized. This is probably due to insufficiently coordinated and managed RTW processes and results in low work stability [13].

For many individuals with mild to moderate TBI, it is a major challenge to maintain employment over time while experiencing somatic, cognitive, and emotional symptoms [12, 14]. Impaired executive functioning, learning, memory, and attention are strongly associated with RTW across a variety of disorders affecting brain function and can result in slowness in work performance; difficulties with learning work tasks; distractibility; and problems with planning, organization, and goal-directed behavior. All of these factors may lead to work failure [13, 14]. However, large-scale literature reviews have documented that rehabilitation programs aimed at teaching patients with mild to moderate cognitive problems strategies to manage and compensate for their problems should be a practice standard [15, 16]. Positive work outcomes following cognitive rehabilitation interventions have been reported in studies on moderate to severe TBI [17], but the evidence is insufficient to draw strong conclusions. Compensatory cognitive interventions typically teach clients strategies to compensate for their cognitive deficits in daily living activities, but vocational rehabilitation is rarely addressed specifically in these TBI programs. Authors of a review published in 2009 found that supported employment (SE), based on long-term support and job skills reinforcement through on-the-job coaching, could overcome the limitations of program-based vocational rehabilitation [18]. Furthermore, authors of a systematic review assessing effective RTW interventions found that involvement of patient and employer and work or workplace accommodations were among the components incorporated in the most effective interventions [19].

To date, only a couple of randomized controlled trials (RCTs) have combined cognitive and vocational rehabilitation/SE for patients with mild to moderate TBI [20, 21]. The only study resembling our present protocol was performed by Twamley et al. [21, 22]. Their 12-week compensatory cognitive rehabilitation intervention (Cognitive Symptom Management and Rehabilitation Therapy [CogSMART]) was offered in addition to SE for U.S. veterans with mild to moderate TBI. All participants were unemployed but wished to return to work. This group was compared with a control group (CG) that received enhanced SE only. Participants assigned to both SE and CogSMART demonstrated significant reductions in postconcussive symptoms and improvements in prospective memory, but there were no effects on RTW. The authors noted that their study was a pilot in need of replication. Moreover, a process evaluation was not performed in their study, and there are significant differences between the United States and Norway regarding the labor market as well as the welfare system. There is a need to explore different stakeholders’ experiences and processes at the workplace in the RTW process. Finally, there are no RCTs in which researchers have examined the cost-effectiveness of vocational rehabilitation following TBI.

### Approaches, hypotheses, and choice of methods

The present study was based on Twamley et al.’s 2014 pilot study that targeted individuals with mild and moderate TBI with persistent cognitive and postconcussive symptoms. The aim of their study was to assess the effect of the CogSMART intervention in combination with SE on improving postconcussive symptoms, neuropsychological performance, quality of life, functional capacity, emotional symptoms, and work participation [21]. Compensatory Cognitive Training (CCT) is a further development of CogSMART [23]. It is a group-based, manualized intervention that includes ten weekly sessions, and it is theoretically based on elements derived from prior cognitive training programs for people with TBI and severe mental illness [21, 23, 24]. The intervention is focused on psychoeducation and compensatory strategy training, and it targets postconcussive symptom management and cognitive symptoms. It is focused on the effect that postconcussive symptoms (such as sleep disturbance, pain, fatigue, headache, tension, and emotional distress) can have on cognitive symptoms and functional recovery. The CCT intervention stresses a biopsychosocial understanding, and it is aimed at educating participants about this complex interrelationship and teaching them stress reduction techniques and strategies to compensate for the functional consequences of the symptoms they are experiencing. The compensatory cognitive strategies target prospective memory, attention and concentration, learning and memory, and executive function [21, 25]. Pilot studies of patients with mild to moderate TBI have demonstrated the efficacy of CogSMART and CCT in improving emotional problems, functional capacity, quality of life, and performance on neurocognitive measures [21–23].

A novel approach to vocational rehabilitation based on the “place-then-train principles” in SE involves support in real-life competitive work settings and is aimed at providing professional services to people with disabilities to help them participate in the competitive labor market [26]. The Five-Step Process describes support activities in the inclusion process: engagement, vocational profiling, job finding, employer engagement, and on-/off-job support. The Individual Placement and Support (IPS) Fidelity Scale is based on eight principles: competitive employment, eligibility based on client choice, integration of rehabilitation and health care services, attention to client preferences, personalized benefits counseling, rapid job search, systematic job development, and time-unlimited and individualized support. SE has not been evaluated in RTW for individuals with TBI in the Norwegian context, but IPS has gained empirical support with positive results in terms of both work inclusion and non-work-related outcomes for people with mental illness [27–29]. SE will be implemented in this RCT in combination with CCT. To determine the feasibility of the proposed interventions and the implementation of procedures in a Norwegian context, a feasibility study will be conducted. In the RCT, the effectiveness of a combined cognitive and vocational rehabilitation intervention compared with treatment as usual (TAU; a nonstandardized rehabilitation provided by a multidisciplinary rehabilitation team) will be evaluated.

The following are the main study hypotheses:

- Combined CCT and SE will result in a faster RTW and better work stability, as well as reduced postconcussive symptoms and improved cognition, than TAU.
- Combined CCT and SE will result in reduced emotional distress and improved quality of life compared with TAU.
- Combined CCT and SE will be a cost-effective alternative compared with the TAU condition.
- Factors related both to the workplace and to the patient’s motivation for RTW will affect the RTW process.

## Methods/design

### Study design

**Fig. 1:**
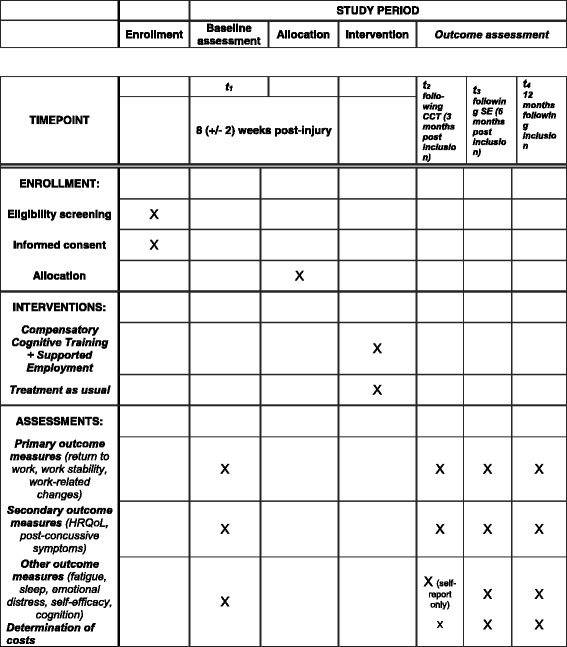
Standard Protocol Items: Recommendations for Interventional Trials (SPIRIT) figure. *CCT* Compensatory Cognitive Training, *HRQoL* health-related quality of life, *SE* Supported employment

**Fig. 2:**
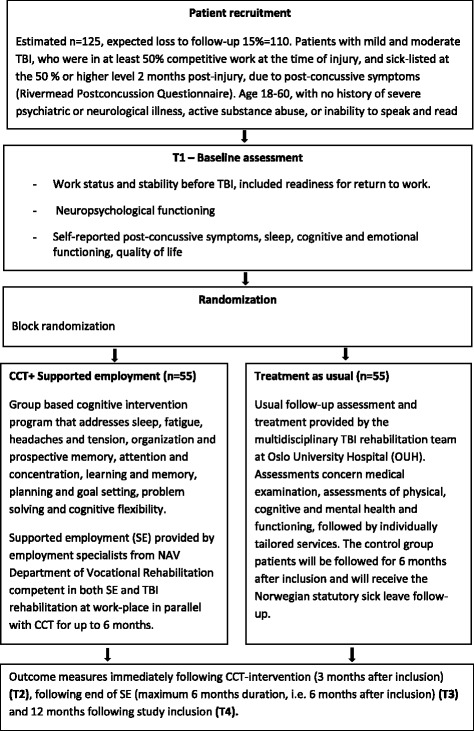
Flowchart for the study protocol. *CCT* Compensatory Cognitive Training, *NAV* Norwegian Labor and Welfare Service, *TBI* Traumatic brain injury

The proposed study will be a parallel-group RCT with a mixed method in design. Based on the Standard Protocol Items: Recommendations for Interventional Trials (SPIRIT), the study flowchart, standard protocol items, and SPIRIT checklist are provided in Figs. 1 and 2, and Additional file 1, respectively. Once included, participants will undergo a baseline assessment of cognitive and emotional status (T1), with further assessments immediately following the CCT intervention (3 months after study inclusion [T2]), following the end of SE (maximum 6 months after inclusion [T3]), and 12 months after study inclusion (T4). All study assessments will be conducted at Oslo University Hospital (OUH). A process evaluation will be performed to explore participants’ experiences with the intervention, as well as individual and workplace-related mechanisms of importance in the RTW process. A qualitative evaluation study regarding patient experiences with CCT is also being planned.

### Study setting

Participants will be recruited from OUH and from general practitioners’ practices. OUH is the trauma referral center for the southeastern region of Norway and has a population base of more than half of the Norwegian population (i.e., 2.9 million). A feasibility study was conducted in the spring of 2017; recruitment for the RCT began in July 2017; and recruitment will continue until the required sample size has been achieved.

### Eligibility criteria

The study population will consist of patients with mild to moderate TBI as assessed by a Glasgow Coma Scale (GCS) score of 10–15, loss of consciousness (LOC) for < 24 h, and posttraumatic amnesia (PTA) for < 7 days [25]. Confirmation of the diagnosis of mild TBI will be done by documenting that acute symptoms adhere to the American Congress of Rehabilitation Medicine’s definition of mild TBI [30]. This will be either extracted from preexisting medical records or established at the time of screening for study eligibility. Patients will be considered eligible for study inclusion if they are employed in a minimum 50% position at the time of injury and are sick-listed at the 50% or higher level because of postconcussive symptoms 2 months postinjury as assessed by the Rivermead Post-Concussion Symptoms Questionnaire [31]. Participants will be aged 18–60 years and reside in Oslo or Akershus County. Patients with a history of severe psychiatric or neurological illness, active substance abuse, or inability to speak and read Norwegian will be excluded.

### Patient characteristics

The following sociodemographic variables will be recorded: age, sex, marital status, living conditions, educational level, description of preinjury employment, workplace, and work stability (days on sickness benefits 1 year prior to the injury). The Readiness for Return to Work scale [32] will be applied at baseline to assess the participants’ perceptions regarding RTW. To measure the impact of the work environment on the RTW process, the Copenhagen Psychosocial Questionnaire (COPSOQ) short version [33] will be applied at baseline. Medical variables include injury characteristics and clinical severity (GCS, LOC, PTA), neuroimaging results, length of hospitalization, medical treatment modalities, postconcussion symptoms, fatigue, and insomnia.

### Outcomes

The primary outcome measures are work participation as measured by the proportion of participants who have returned to work at 12-month follow-up and length of time before return to work (in days), work productivity (hours worked, work-related changes [i.e., reduced productivity, increased supervision, work content changes], and work stability [i.e., sickness absence after initial RTW and throughout the study period]). To provide descriptive information and group comparisons, an IQ estimate (Vocabulary, Similarities, Block Design, and Matrix Reasoning subscales of the Wechsler Adult Intelligence Scale–Fourth Edition) will be included at baseline (T1) only [34].

Postconcussive symptoms and quality of life will be used as secondary outcome measures at all time points (T1–T4), whereas outcome will also be measured in the domains of fatigue, sleep, emotional distress, self-efficacy, and cognition. The instruments that will be applied are listed in Table 1. Neuropsychological evaluation will not be conducted at T2.

**Table 1:** Secondary and other outcome measures

Outcome measure	Measures
Secondary outcomes	
Postconcussive symptoms	Rivermead Post-Concussion Symptoms Questionnaire (RPQ) [31]
Quality of life	EuroQol five dimensions (EQ-5D) [48]
	Quality of Life After Brain Injury instrument (Qolibri) [49]
Other outcome measures	
Fatigue	Fatigue Severity Scale (FSS) [50]
Sleep	Insomnia Severity Index (ISI) [51]
Self-reported cognitive functioning	Cognitive Failures Questionnaire (CFQ) [52]
Emotional functioning (anxiety and depressive symptoms, posttraumatic stress symptoms, and self-efficacy)	The Patient Health Questionnaire (PHQ-9) [53]
	Generalized Anxiety Disorder seven-item (GAD-7) scale [54]
	Posttraumatic Symptom Scale (PTSS-10) [55]
	General Self-Efficacy Scale (GSS) [56]
Neuropsychological test measures	
Learning and memory	California Verbal Learning Test–Second Edition (CVLT-II) [57]
Prospective memory	Memory for Intentions Screening Test (MIST) [58]
Processing speed/executive function	Color Word Interference Test from the Delis-Kaplan Executive Function System (D-KEFS) [59]
	Trail Making Test (TMT) from the Delis-Kaplan Executive Function System (D-KEFS) [59]
	Coding from the Wechsler Adult Intelligence Scale–Fourth Edition (WAIS-IV) [34]
	Ruff 2 and 7 Selective Attention Test [60]
	Modified Six Elements Test from the Behavioral Assessment of the Dysexecutive Syndrome (BADS) (to be applied at T4 only) [61]
Validity	Forced Choice Recognition index from the CVLT-II [57]

### Process evaluation: qualitative perspective on RTW process

The aim of the qualitative process evaluation is to explore features of the “train and maintain” elements that take place at the workplace during the RTW process (i.e. types of support, as well as if and how the employment specialists manage to generate partnerships with employers for job adjustments and adapt work, job development, and job carving to fit the needs of employees with TBI). Additionally, we will assess how risks, challenging behavior, and conflicts are dealt with, as well as how natural internal company support and increased inclusion competencies within the workplaces may be developed. In line with theory [35], we assume that increased practical knowledge of opportunities and obstacles for work inclusion at the workplace generates increased commitment among the relevant stakeholders. This knowledge and learning process in the work organization may increase the possibility for successful RTW and job tenure. Furthermore, via the qualitative evaluation process, we will seek to identify which managerial styles are most effective in creating secure work environments and promoting successful RTW processes for the targeted population.

The process evaluation will be based on semistructured interviews in individual RTW case processes. Each case process will include three informant groups: participant, workplace employer, and employment specialist (intervention group only) or supervisor at the local office of the public Norwegian Labor and Welfare Service (NAV) (CG only). Interviews will be conducted at two different time points in each case process: 1 month and 12 months following RTW. The interviews will provide an empirical base for additional knowledge on job content and skill requirements (i.e., possibilities for adapted/alternative work tasks); work environment quality (i.e., demands, control, support); the role of management as well as types and features of work organization (i.e., division of tasks, specialization, interdependencies), and what kind of external support is needed (i.e., the employment specialists’ contributions that influence job match achievement and the sustainability of the work relationship).

The contents of the interview questionnaires are developed on the basis of selected elements from the COPSOQ [33], the Multifactor Leadership Questionnaire [36], and the SE Five-Step Process and IPS Fidelity Scale [37, 38]. In the interviews in both the intervention and control groups, we will seek to achieve the following:

- Identify the employment specialist’s methods as well as cooperation between the employment specialist and the CCT team, the employment specialist and the employer, and the employment specialist and the person with TBI
- Identify attributes and workplace conditions (e.g., work tasks/production, internal company training systems, work environment, and management factors) that may influence the RTW process
- Identify work organizational indicators for successful RTW and job stability
- Identify significant changes in individual inclusion and exclusion processes at the work organization level
- Identify effective managerial styles that promote RTW

### Sample size

Regarding RTW, an OR of 2.0 between the Compensatory Cognitive Training–Supported Employment (CCT-SE) and CG is regarded as the smallest relevant clinical and societal OR. Thus, the required total sample size calculated using G*Power is 110 (i.e., 55 persons in each group; α = 0.05, power level of 0.80) [39]. With an estimated loss to follow-up of 15% [40], 125 participants will be required. On the basis of an ongoing TBI study [41], we assume that this will be achievable within 12–18 months. An OR of 2.0 is equivalent to a 33% absolute difference in employment status between the two groups. According to Twamley et al. [21], 50% of patients attained competitive work at 12-month follow-up. If the proportion of employed patients in the CG is 50% at 12-month follow-up, the proportion for the intervention group in this study will be expected to be 83% or above on the basis of the given estimation.

Two strategic samples will be drawn for the process evaluation, comprising 40 cases from the intervention group and 20 from the CG. It is a goal to achieve a heterogeneous sample with variation in severity of TBI, sex, age, type of job and workplace, company size, industry, and sector.

### Randomization

A permuted block randomization sequence will be generated by an independent statistician prior to the start of the trial. Eligible patients who consent to study participation will be randomly allocated in a 1:1 ratio in each block to receive either the study intervention or TAU (CG). An investigator who is independent of the patient screening process will be responsible for allocating the patients to the study conditions. Blinding of the patients and rehabilitation professionals is not possible, but the outcome assessors will be blinded to study allocation.

### Study interventions

#### CCT-SE intervention

The CCT-SE intervention will comprise a 10-week manualized group intervention that includes weekly CCT group sessions with three to seven participants, which will be provided by a psychologist at OUH. CCT includes psychoeducation, strategy training, and establishment of new habits in several domains. Patients learn about the natural course of postconcussive symptoms and are introduced to sleep hygiene and stress reduction techniques. Compensatory cognitive strategies are taught regarding organization and prospective memory (task management), attention and concentration (during tasks and social interaction), planning and goal setting, learning and memory (internal and external strategies), and executive function (problem solving and self-monitoring). The CCT manual has been translated into Norwegian and adapted to Norwegian conditions in collaboration with the original author of the manual (Twamley [21, 42]). The Norwegian user organization (Personskadeforbundet LTN) has participated in the translation process [43].

The vocational part of the intervention is based on SE principles and will be provided by three trained employment specialists from the NAV Department of Vocational Rehabilitation. For the purpose of this study, the participants will receive vocational intervention for a maximum of 6 months. A main emphasis will be on stages 1 (client engagement), 4 (employer engagement), and 5 (on- and off-the-job support) of the SE Five-Stage Process [38], because all participants will be in regular employment at the time of injury. The initial contact with the participant will be focused on establishing a trustful relationship between the employment specialist and the participant. The employment specialists will use the approach of “discovery,” a process for involving the participant in his/her own RTW process. The next step is mapping the patient’s resources, limitations, and work tasks, as well as establishing common goals between the employment specialist and the participant. The following sessions will be customized to the employee’s needs and may include consultations, guidance and advice, learning/training, work task adaptations, and assistive technology. The sessions may also include the employer and the supervisor at the local NAV office if considered beneficial. The vocational intervention will be integrated with standard Norwegian statutory sick leave follow-up. The International Classification of Health Interventions (ICHI) will be applied for the standardization and documentation of the individualized interventions [44]. ICHI is a tool developed by the World Health Organization for reporting and analyzing health interventions and covers interventions carried out by a broad range of health care providers, including acute care, postacute care, and rehabilitation, as well as assistance with functioning, health prevention, and public health matters. ICHI is still under development, and the last published version is denoted as alpha version 2. The classification will be ready for operational use during the study period.

For the CCT-SE, three NAV employment specialists in the project will follow one group program each to become well acquainted with CCT content and ensure implementation of strategies and compensatory techniques at the workplace. Continuous cooperation between the CCT team, employee, and SE personnel will be emphasized. Employment specialists have participated in formalized postgraduate SE education at Oslo and Akershus University College of Applied Sciences (HiOA). The content of the education is based on the SE Five-Stage Process and the SE Fidelity Scale [37, 38]. Supervision of the employment specialists will be provided by HiOA, with special attention given to discovery, working with employers, on- and off-the-job training, and ongoing support.

The two doctoral candidates in the project will be responsible for provision of the CCT intervention. They are both experienced psychologists and will work in close collaboration with their doctoral program supervisors and the intervention developer (E. W. Twamley). The feasibility study will ensure adequate training and provide an opportunity to make necessary adjustments to the Norwegian version before inclusion in the RCT.

#### TAU

The CG will receive TAU, which includes follow-up assessment and treatment provided by the multidisciplinary TBI rehabilitation team at OUH. The team consists of six rehabilitation professionals, thus fulfilling requirements for complex rehabilitation [45]. Patients will undergo a medical examination and assessment of physical, cognitive, and mental health and functioning, followed by individually tailored services. The CG will be followed for 6 months after inclusion. These patients will also receive the Norwegian statutory sick leave follow-up, and the treatment received will be registered and mapped according to the ICHI.

### Statistical analysis

Descriptive statistics will be used to describe the baseline and injury characteristics of the variables related to participants and services. The *t* test will be used to analyze between-group mean comparisons for normally distributed continuous data, and the Mann-Whitney *U* tests will be used to analyze skewed data. For the primary outcome measures, a logistic regression model will be used to compare the proportions of participants returning to work at T4 in the CCT-SE and TAU groups, adjusting for other potential confounders. In addition, linear regression analysis will be applied to compare the difference in the mean length to RTW between the intervention groups at T4, adjusting for other potential confounders. For the secondary outcome measures, repeated measures analysis of variance will be used with time (T1–T4) as the repeated-measures factor and group (CCT-SE and TAU) being a between-group factor to test whether the CCT-SE intervention has a beneficial effect compared with TAU on RTW, symptoms, and functioning. The intention-to-treat principle will be applied for all proposed analyses.

#### Process evaluation analysis

Each semistructured interview will be audio-recorded and last approximately 60 minutes. After the interview, the researchers will complete a table describing the main topics that emerged. Each interview will be summarized into keywords and coded into the table. Each case process will be coded into one overarching table, including different informant groups’ perspectives within the same case over time. Finally, all cases will be analyzed thematically.

#### Health economic analysis

Information concerning costs will be gathered at follow-up (T2–T4) using a cost registration form. For the calculation of the total costs, direct health care costs (i.e., health care provider costs), direct nonhealth costs (i.e., costs of informal health care), and indirect costs (i.e., loss of paid and unpaid work productivity) will be determined. Costs of interventions and patient income will be calculated in Norwegian kroner.

Cost-effectiveness and cost-utility analyses will be performed to determine the cost-effectiveness of the intervention. The analysis will be based on the effect of the intervention on RTW/work participation and effect on functioning, including health-related quality of life (HRQoL). First, we will calculate the economic benefit as a result of the employment effect of the intervention (income and cost of intervention) as compared with the CG. Second, the cost of the intervention will be seen in terms of health benefits (improved HRQoL). Using standardized conversion tools, it is possible to convert health benefits into an index of HRQoL as measured by the EuroQol five dimensions questionnaire (EQ-5D). With this analysis, we can compare quality of life in the intervention group and the CG. Standard discounting will be performed for both costs and outcomes together with sensitivity and uncertainty analysis. Full- or part-time work will be accounted for.

### Ethics and dissemination

The study has been presented to and approved by the Norwegian Regional Committee for Medical and Health Research Ethics (REK) (REK number 2016/2038). The project will be conducted according to the ethical guidelines of the Helsinki declaration [46]. Information about the study will be presented to the patients in written and oral form. Written informed consent will be obtained, and the right to withdraw from the project at any time without any explanation necessary will be emphasized. We consider the randomization procedure to be ethically acceptable. All data will be unidentifiable when sharing between partners, and personal data will not be identifiable in the analysis or presentations.

The study will be conducted in close collaboration with the user organization [43]. The user organization is represented in the management committee and has had an active role in the translation and adaptation of the cognitive intervention manual to the Norwegian setting.

The anonymized quantitative data will be stored in the database on the research server at OUH. In the qualitative part of the study, additional informed consent will be obtained from workplace managers, employment specialists, and supervisors at the local NAV office who will be interviewed. The qualitative data (the audio recordings of the interviews) will be properly stored in controlled access folders on an HiOA research server. Both tapes and transcripts will be kept locked at the Work Research Institute/HiOA. All data will be securely contained for 5 years after the end of the project.

The trial report and other dissemination documents will be written according to the Consolidated Standards of Reporting Trials (CONSORT) statement to facilitate complete and transparent reporting and aid in critical appraisal and interpretation [47]. The dissemination plan reflects the research communities involved in this multidisciplinary project. We aim to publish reports of the project in journals of neurology, neuropsychology, brain injury rehabilitation, occupational research, and social sciences. Experiences with and results of the project will also be disseminated in relevant expert forums, national and international meetings, conferences, popular scientific journals, and reports. The results will also be shared with the user organization and its members through their communication channels in print and on the Internet.

## Discussion

This project is highly innovative by involving cross-sectoral partnerships (between specialized health care and research services, the labor and welfare system, and work and social scientist milieus) in a well-controlled RCT on cognitive and vocational rehabilitation after TBI. The project results can inform decisions and ultimately labor and welfare system practice. Because cognitive difficulties and challenges in RTW are not limited to TBI, this study has potential relevance to other patient groups whose cognitive symptoms complicate work participation. The RCT will provide knowledge about the cost-effectiveness of the treatment program. The project will have an impact on knowledge of the “train and maintain” aspects of support systems and businesses dealing with sick leave and RTW, as well as on the further development of the SE approach, especially concerning the role of the employment specialist at the workplace. Because TBI tends to affect young people, there is considerable potential societal monetary gain, given that the intervention results in faster and more stable long-term RTW. Thus, the project can serve as a benchmark study regarding the efficacy of combined cognitive rehabilitation and SE efforts.

### Limitations

The present protocol has limitations that should be addressed. First, this is a pragmatic clinical trial in which the nature of the interventions prevents blinding of participants and therapists. Furthermore, outcome assessment will be performed by personnel unaware of group assignment. Second, the participants will be allocated to one of two groups, TAU or CCT combined with SE, potentially making it difficult to tease apart the active ingredients of the CCT-SE intervention. However, as mentioned previously, what makes this study innovative is the combination of rehabilitation and vocational science perspectives, in addition to strong cross-sectoral collaboration between specialized health care services and the welfare system. If we had decided to include a CG with traumatic injury but without head trauma, it would have been possible to identify nonspecific effects of traumatic injury that may contribute to symptoms and lasting functional impairment. The grant provided to us unfortunately prevents us from implementing this. Furthermore, the main aim of this study is to affect work participation through a combination of cognitive remediation and SE. For this purpose, a TAU CG seems appropriate because etiology and causation will be comparable across groups.

A third possible limitation is the risk of nonadherence to the interventions and losing patients to follow-up (i.e., risk of dropout). To facilitate study adherence and keep the dropout rates as low as possible, the research team will be well-trained, perform outreach, and be flexible with respect to timing of the intervention. Last, this trial is taking place in the southeastern region of Norway, and participants might not be representative of the whole population of Norway. It should also be mentioned that this is a single-center trial, which could potentially limit external validity. However, we are confident that the results could be generalizable, because more than half of the Norwegian population resides in this region.

### Trial status

This is protocol version 1.0. A feasibility study including six patients has been performed and concluded in July 2017. The results of the feasibility study are being prepared for publication. No major changes to the protocol were made as a result of the feasibility study. Recruitment and randomization of participants for the main study commenced in July 2017 and will end when we have enrolled the estimated sample size (approximately in November/December 2018).

## Additional file

Additional file 1: SPIRIT checklist. (PDF 128 kb)

## Abbreviations

CCT: Compensatory Cognitive Training
CCT-SE: Compensatory Cognitive Training–Supported Employment
CG: Control group
CogSMART: Cognitive Symptom Management and Rehabilitation Therapy
CONSORT: Consolidated Standards of Reporting Trials
COPSOQ: Copenhagen Psychosocial Questionnaire short version
CVLT-II: California Verbal Learning Test–Second Edition
EQ-5D: EuroQol five dimensions questionnaire
GCS: Glasgow Coma Scale
HiOA: Oslo and Akershus University College of Applied Sciences
HRQoL: Health-related quality of life
ICHI: International Classification of Health Interventions
IPS: Individual Placement and Support
LOC: Loss of consciousness
NAV: Norwegian Labor and Welfare Service
OUH: Oslo University Hospital
PTA: Posttraumatic amnesia
RCT: Randomized controlled trial
REK: Norwegian Regional Committee for Medical and Health Research Ethics
RTW: Return to work
SE: Supported employment
SPIRIT: Standard Protocol Items: Recommendations for Interventional Trials
TAU: Treatment as usual
TBI: Traumatic brain injury
WAIS-IV: Wechsler Adult Intelligence Scale–Fourth Edition
